# Chronic polytherapy after myocardial infarction: the trade-off between hospital and community-based providers in determining adherence to medication

**DOI:** 10.1186/s12872-021-01969-9

**Published:** 2021-04-14

**Authors:** Mirko Di Martino, Michela Alagna, Adele Lallo, Kendall Jamieson Gilmore, Paolo Francesconi, Francesco Profili, Salvatore Scondotto, Giovanna Fantaci, Gianluca Trifirò, Valentina Isgrò, Marina Davoli, Danilo Fusco

**Affiliations:** 1 Department of Epidemiology, Lazio Regional Health Service, ASL Roma 1, Via Cristoforo Colombo, 112, 00147 Rome, Italy; 2 Management and Healthcare Laboratory, Scuola Superiore Sant’Anna, Pisa, Italy; 3 Epidemiology Unit, Regional Health Agency (ARS) of Tuscany, Florence, Italy; 4 Department of Epidemiologic Observatory, Health Department of Sicily, Palermo, Italy; 5 Department of Biomedical and Dental Sciences and Morpho-Functional Imaging, University of Messina, Messina, Italy

**Keywords:** Myocardial infarction, Adherence to chronic poly-therapy, Geographic variation, Hospital of discharge, Community-based healthcare providers

## Abstract

**Background:**

The benefits of chronic polytherapy in reducing readmissions and death after myocardial infarction (MI) have been clearly shown. However, real-world evidence shows poor medication adherence and large geographic variation, suggesting critical issues in access to optimal care. Our objectives were to measure adherence to polytherapy, to compare the amount of variation attributable to hospitals of discharge and to community-based providers, and to identify determinants of adherence to medications.

**Methods:**

This is a population-based study. Data were obtained from the information systems of the Lazio and Tuscany Regions, Italy (9.5 million inhabitants). Patients hospitalized with incident MI in 2010–2014 were analyzed. The outcome measure was medication adherence, defined as a Medication Possession Ratio (MPR) ≥ 0.75 for at least 3 of the following drugs: antiplatelets, β-blockers, ACEI/ARBs, statins. A 2-year cohort-study was performed. Cross-classified multilevel models were applied to analyze geographic variation. The variance components attributable to hospitals of discharge and community-based providers were expressed as Median Odds Ratio (MOR).

**Results:**

A total of 32,962 patients were enrolled. About 63% of patients in the Lazio cohort and 59% of the Tuscan cohort were adherent to chronic polytherapy. Women and patients aged 85 years and over were most at risk of non-adherence. In both regions, adherence was higher for patients discharged from cardiology wards (Lazio: OR = 1.58, *p* < 0.001, Tuscany: OR = 1.59, *p* < 0.001) and for patients with a percutaneous coronary intervention during the index admission. Relevant variation between community-based providers was observed, though when the hospital of discharge was included as a cross-classified level, in both Lazio and Tuscany regions the variation attributable to hospitals of discharge was the only significant component (Lazio: MOR = 1.30, *p* = 0.001; Tuscany: MOR = 1.31, *p* = 0.001).

**Conclusion:**

Adherence to best practice treatments after MI is not consistent with clinical guidelines, and varies between patient groups as well as within and between regions. The variation attributable to providers is affected by the hospital of discharge, up to two years from the acute episode. This variation is likely to be attributable to hospital discharge processes, and could be reduced through appropriate policy levers.

**Supplementary Information:**

The online version contains supplementary material available at 10.1186/s12872-021-01969-9.

## Introduction

Patients with previous myocardial infarction (MI) are at increased risk of repeated MI and death. Current guidelines on secondary prevention following MI recommend the use of combined therapy, composed of platelet aggregation inhibitors (antiplatelets), beta-blocking agents (β-blockers), agents acting on the renin-angiotensin system (ACEI⁄ARBs) and HMG-CoA reductase inhibitors (statins) [1, 2]. The benefits of chronic polytherapy in reducing readmissions (relative risk reduction up to 77%) and death (relative risk reduction up to 65%) have been clearly shown [3–8]. However, real-world evidence shows poor adherence to chronic polytherapy [9, 10]; poor adherence to medical treatment severely compromises patient outcomes and increases mortality [11]. Moreover, large geographic variation has been observed in adherence to evidence-based pharmacotherapy. This heterogeneity raises concerns over access to optimal care [12, 13]. To improve medication adherence, the multifactorial causes of this phenomenon must be understood. According to the World Health Organization, these causes fall into three broad categories: patient related factors, physician related factors, and health system related factors [11]. Currently, the majority of initiatives to increase adherence are focused on out-of-hospital settings delivering ongoing support from a range of health professionals, with limited focus on hospital settings [14, 15]. This is in line with the broader policy and practical focus on out-of-hospital support for individuals with chronic conditions. However, existing evidence from the scientific literature does not allow quantification of the gaps between clinical practice and guidelines attributable to community-based healthcare providers—such as General Practitioners (GPs), Local Health Districts (LHDs) and Local Health Units (LHUs)—and to discharging hospitals. New methods for quantifying the contributions of different system components (e.g., community-based providers, or hospitals of discharge) would be very useful to define the strategic focus for more targeted interventions aimed at improving adherence to guidelines, and thereby equity in healthcare.

### Objectives of the study

The objectives of this study are: (1) to measure adherence to chronic polytherapy after MI in a real-world setting; (2) to quantify and compare the impacts on medication adherence attributable to community-based healthcare providers or hospitals of discharge; (3) to identify patients’ determinants of medication adherence; (4) to summarize relevant policy and practice responses which could support improved medication adherence.

## Materials and methods

### Settings and data sources

This is a real-world, retrospective follow-up study, partially funded by the Italian Ministry of Health, and approved by the ethics committee [16]. Study subjects were recruited from individuals registered with the Local Health Units of two Italian regions: Lazio and Tuscany (a total of over 9.5 million residents). LHUs are administrative bodies of the regions, responsible for fulfilling the tasks of the National Health System (NHS) in a determined area. Each LHU is organized into LHDs comprising a defined group of GPs coordinated by a district director. The LHDs directly provide primary care services related to health and social-health activities at the local level. Patients’ characteristics were retrieved from the regional health information systems that include mortality, hospital admissions, and drug claims data. Details of the individual information systems are reported in Additional file 1.

### The study cohort

All patients aged 35–100 years, discharged alive from hospital with a diagnosis of MI between 1 January 2010 and 31 December 2014 (the enrollment period) were recruited in the study. A MI was defined as a primary diagnosis of International Classification of Diseases Ninth Revision Clinical Modification (ICD-9-CM) codes 410.xx or as a primary diagnosis of MI-related condition along with a secondary diagnosis of 410.xx (see Additional file 1). The first hospitalization during the enrollment period fulfilling selection criteria was considered the index admission. The cohort is composed of only the incident cases of MI: patients with hospitalizations for MI related causes such as percutaneous coronary intervention (PCI), bypass (CABG), ischemic heart disease, or surgery of heart and great vessels in the 9 years before the index admission were not considered eligible for the study. To avoid problems tracing subjects in regional health information systems, only patients present in the regional health assistance file for the entire analysis period were included in the cohort. Subjects with a duration of index admission > 95th percentile were excluded from the analyses as they were considered “statistical outliers”, probably representing particularly complex cases.

### Follow-up

For each patient, the follow-up period for measuring drug adherence starts on the day following discharge from the index admission. The minimum qualifying follow-up period was 30 days, determined a priori as the minimum time enabling a consistent estimate of adherence to polytherapy [17]. The maximum duration of follow-up is two years, and ends earlier when one of the following “censorship episodes” occurs: the patient’s death, or a hospital admission for any cause after the discharge of index admission. This last “criterion of censorship” is due to the need to evaluate the net impact of the discharging hospital following the acute episode, without the potential interference of further hospitalizations.

### Drug exposure: the adherence to medications

For each patient, the prescriptions of antiplatelets, β-blockers, ACEI/ARBs and statins delivered during the follow-up period were collected and analyzed (see Additional file 1 for ATC codes). All drugs in this study were included in the patients’ health care plans and were equally available to all residents, in accordance with the universal health care coverage provided to residents of Italy. Adherence to each drug for secondary prevention of MI was measured through the medication possession ratio (MPR), which is calculated as the ratio between the number of days of medication supplied during the follow-up, on the basis of defined daily doses (DDDs), and the number of calendar days in the follow-up [18]. The patient was classified as adherent to the prescribed therapy for a given drug if the proportion of days covered by the therapy is greater than or equal to 75% of the follow-up period (MPR ≥ 0.75). Adherence to chronic polytherapy was defined as an MPR ≥ 0.75 for at least three of the four evidence-based (EB) drugs.

### Data structure and statistical analysis

#### The hierarchical structure of data

Data associated with community-based healthcare providers (LHUs, LHDs, GPs) have a hierarchically organized structure, in which each lower-level unit belongs to one and only one higher-level unit. The study of adherence to polytherapy was therefore initially carried out by performing logistic multilevel analysis at four levels, considering the nesting of each patient within their own GP, who in turn is a member of a particular health district, which belongs to a specific LHU. This approach is able to ‘decompose’ the total variability in adherence to EB drugs in distinct and additive components attributable to each of the different community-based providers. A further level of analysis, the hospital of discharge, was added to the hierarchical structure just described. This introduction makes the organization of data more complex, transforming the hierarchical structure into a cross-classified structure in which elementary units are classified according to two or more factors that are not hierarchically ordered [19]. In this way, cross-classified logistic multilevel modelling was performed in order to analyse geographic variation—measuring and comparing the proportion of variability attributable to hospitals of discharge and to community providers.

#### The median odds ratios

The variance components of the multilevel models are expressed in terms of Median Odds Ratio (MOR), a measure that quantifies the variability between cluster—in this case, healthcare providers. Another important feature of MORs is that they express the variability between micro-clusters belonging to the same macro-cluster, e.g., the variability between GPs belonging to the same LHD. MORs always are equal to or greater than 1.00. A MOR equal to 1.00 indicates no variability between clusters; as the variability between groups increases, the value of the measure increases [20]. The MOR was estimated for each type of healthcare provider. It is worth noting that, in this framework, MORs constitute a system of weights which are directly proportional to the impact of the corresponding provider on adherence to chronic polytherapy [17]. MORs were estimated with controlling for patient characteristics, to ensure that different compositions of patients within groups (in terms of age, comorbidities or severity of MI) did not influence estimates of variance.

#### Determinants of patient adherence

The introduction of the first-level covariates in the model allowed the identification of determinants of adherence to EB drugs. Factors included in the analysis were: patient demographic characteristics (gender, and age at discharge); variables related to the index admission, which can be considered as a proxy for the severity of MI (length of hospital stay, presence of PCI or CABG intervention, heart or large vessels surgery, diagnosis of ‘other forms of ischemic heart disease’ and type of MI, defined as ‘ST-elevation myocardial infarction’ or ‘non-ST-elevation myocardial infarction); the use of the four drugs for secondary prevention during the 12 months prior to the index admission (defined as at least two prescriptions); the ward of discharge; and 21 comorbidities retrieved from hospital records for both the index admission and the 9 previous years (see Additional file 1 for details on comorbidities and their selection algorithm). The analyses were performed separately for each of the two regions. MORs, Odds Ratios (ORs), 95% confidence intervals (95% CIs) and p-values are reported.

## Results

### The study cohort

**Fig. 1:**
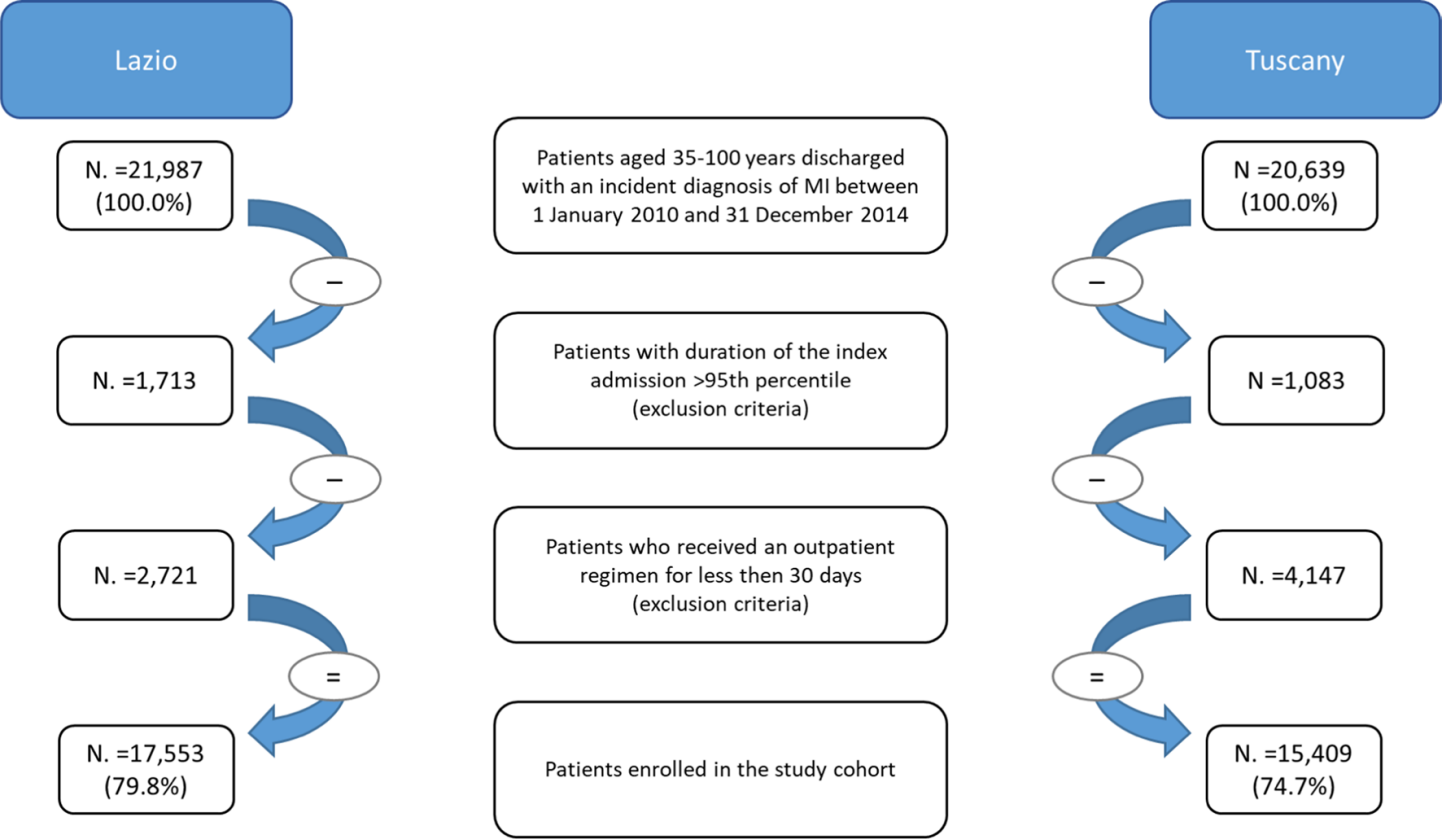
Flow-chart

After applying the exclusion criteria described above, 17,553 subjects in the Lazio region and 15,409 in Tuscany region were enrolled in the cohort of patients with incident diagnosis of MI (Fig. 1). The median follow-up time was 530 days for patients in both regions. Men represented 69% of the Lazio cohort and 63% of the Tuscany cohort. The Tuscan cohort was on average older than Lazio: the mean age was 67 ± 13 for men and 76 ± 12 for women in the Tuscany region, 64 ± 12 for men and 73 ± 12 for women in Lazio. About 76% of subjects with a first diagnosis of MI in the Lazio region, and 75% in the Tuscany region, had at least one co-existing disease. The most common comorbidities, although with different prevalences, were the same in both cohorts (Table 1): hypertension, lipid metabolism disorders, conduction disorders and cardiac dysrhythmias, diabetes, and heart failure. Regarding the index admission, ST-Elevation Myocardial Infarction (STEMI) occurred in 50% of Lazio patients (8772 subjects) and 48% of Tuscany patients (7396 subjects), whereas percutaneous coronary intervention was performed in 72% and 68% of Lazio and Tuscany patients, respectively. The discharging ward was a non-cardiology ward in 5% of cases in Lazio and 12% of cases in Tuscany.

**Table 1:** The most common comorbidities, by region

Comorbidities (%)	Lazio	Tuscany
Hypertension	48.32	40.00
Lipid metabolism disorders	22.44	21.81
Conduction disorders and cardiac dysrhythmias	17.80	16.61
Diabetes	17.58	16.94
Heart failure	8.43	21.04

### Adherence to medications

Data on adherence to medications are reported in Table 2. The individual EB drug with lowest adherence for both Lazio and Tuscany cohorts was the β-blocker (51% and 46% respectively), while those with the highest proportions of adherent patients were antiplatelets in the Tuscany region (79%, compared to 74% in Lazio) and statins in the Lazio region (77%, against 74% in Tuscany). Finally, 65% of the patients of the Lazio cohort and 61% of the Tuscany cohort were adherent to ACEI/ARBs. After the age of 70, adherence decreased for each of the drugs considered. The most dramatic decrease was found, in both populations, for statins: in the transition from the 74–80 age class to that of older patients, adherence to statins decreased by 20 percentage points in Lazio and by 30 percentage points in Tuscany.

**Table 2:** Adherence (%) to individual EB drugs, by age group and region

Age group	β-blockers	ACEI/ARBs	Statins	Antiplatelet
*Lazio*				
35–54	54.20	59.35	82.20	73.14
55–69	54.24	67.50	83.43	75.66
70–84	49.35	69.28	74.35	75.72
85+	38.06	56.15	54.02	67.54
Total	51.09	65.44	77.41	74.43
*Tuscany*				
35–54	52.96	53.28	82.79	79.90
55–69	51.85	64.39	83.72	81.34
70–84	45.48	65.93	75.03	79.16
85+	32.16	50.79	44.75	70.24
Total	46.32	61.12	73.80	78.46

ACEI/ARBS: agents acting on the renin-angiotensin system

**Fig. 2:**
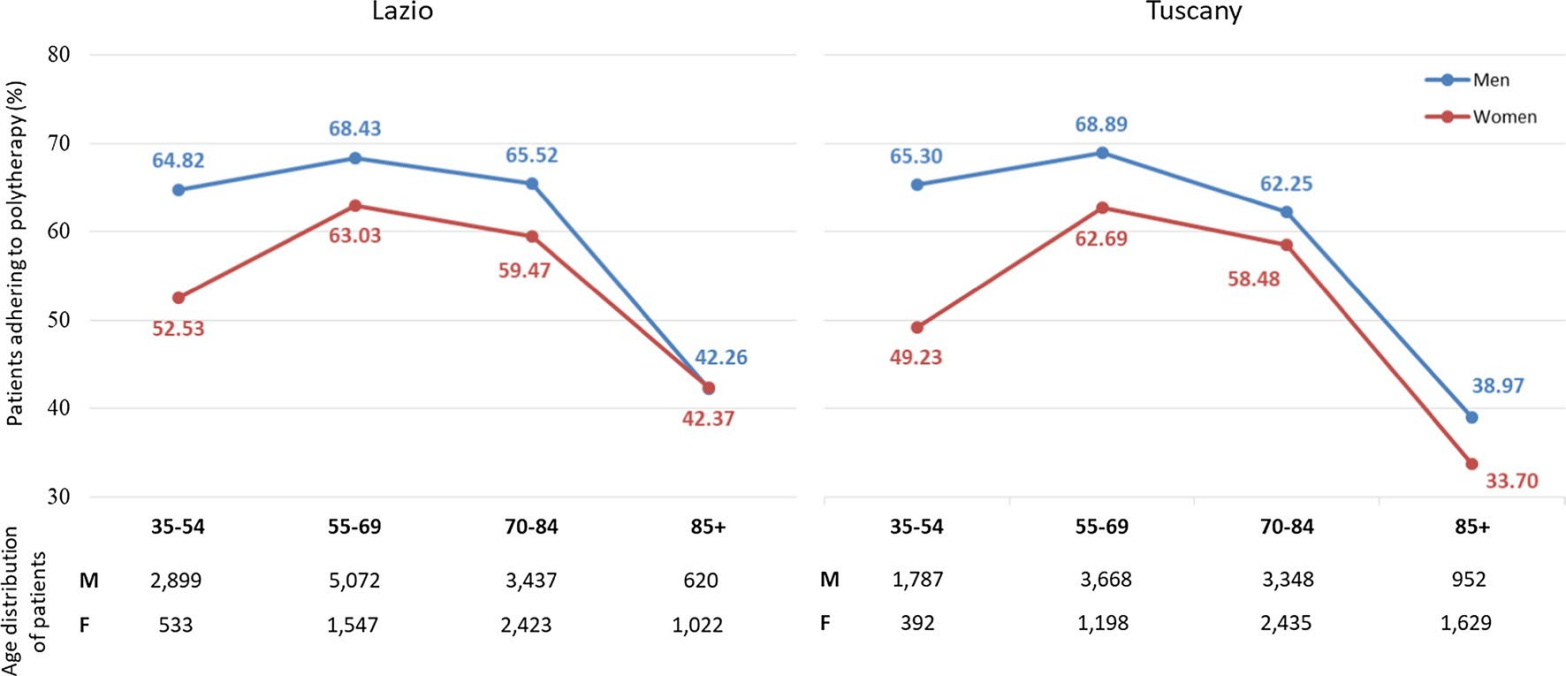
Proportion of patients adhering to polytherapy, by gender and age group

Overall, 63% of Lazio patients and 59% of Tuscany patients where adherent to polytherapy, where adherence is considered as MPR ≥ 0.75 for at least three of the four therapies. The relationship between patient age and adherence to polytherapy is non-linear. As shown in Fig. 2, the curve of adherence as a function of patient age assumes an inverted U-shape. Initially, adherence to polytherapy increases in the transition from the youngest age group to 55–69 years. After the age of 70, the proportion of adherent patients began to decrease, with a nadir in the class of older patients at 85 years and over. Although female adherence to polytherapy was consistently lower than male, both sexes followed the same trend associated with age. With increasing age, a gradual reduction in gender differences was noted. This reduction was particularly evident for the Lazio region, where the proportion of adherent patients was almost equal in men and women aged 85 years and over.

### Determinants of adherence to polytherapy

**Fig. 3:**
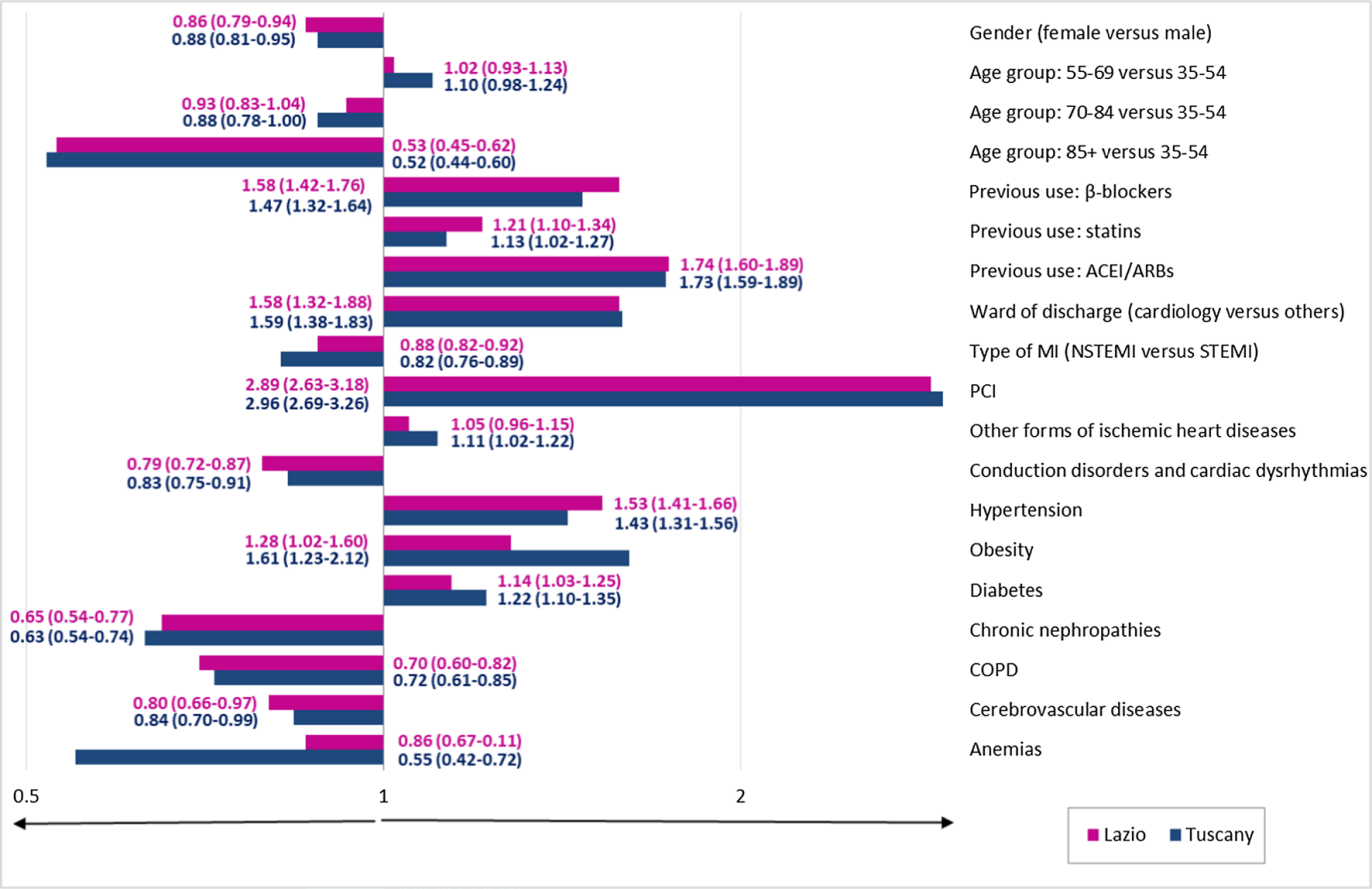
Predictors of adherence to polytherapy. Next to each bar is the value of the Odds Ratio and the relative 95% Confidence Interval. Only statistically significant predictors for at least one region were included in the figure

Figure 3 shows the predictors of adherence to polytherapy. Only those covariates statistically significant for at least one region were included in the graph. The ORs related to the same variable always had the same ‘direction’ in both regions, though with different intensities. For women and older patients, the establishment of a correct therapeutic regimen was more difficult. From the index admission, patients affected by ST-elevation MI were more adherent to polytherapy than patients with non-ST-elevation (NSTEMI). Moreover, presence of PCI surgery was the predictor most associated with medication adherence, both in Lazio (OR = 2.89, 95% CI = 2.63–3.18) and Tuscany regions (OR = 2.96, 95% CI = 2.69–3.26). It is worth noting that patients discharged from cardiology wards had higher levels of adherence than those discharged from other wards (Lazio: OR *cardiology versus others* = 1.58, 95% CI = 1.32–1.88; Tuscany: OR = 1.59, 95% CI = 1.38–1.83). The previous use of individual EB medications, with the exception of antiplatelet drugs, was associated with higher levels of adherence to chronic polytherapy. Some concomitant cardiovascular diseases or risk factors, such as hypertension, diabetes and obesity, increased the likelihood of medication adherence after MI.

### Variation among healthcare providers

**Fig. 4:**
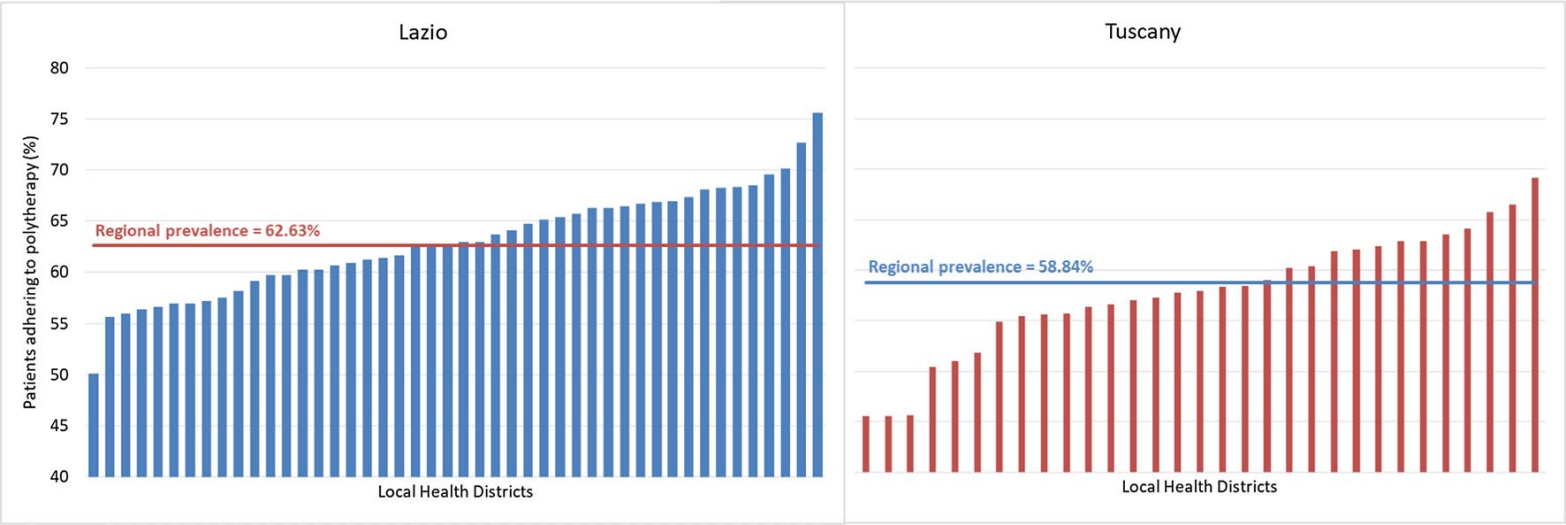
Adherence to polytherapy by local health district. Only Local Health Districts with at least 30 patients are displayed.

Adherence to chronic polytherapy was extremely variable among community-based providers. The notable variability among LHDs in the percentage of adherent patients is shown in Fig. 4; percentages ranged from 50 to 76% in Lazio, in Tuscany from 46 to 69%. In order to quantify how much of the total variability was attributable to the different healthcare providers, we analyzed the “hierarchies” in the healthcare system, which include, for Lazio and Tuscany regions respectively: general practitioners (4226 and 2589), LHDs (46 and 33), LHUs (10 and 12) and—when considering the cross-classified model—hospitals of discharge (82 and 61). In Table 3, MORs attributable to the different community-based providers are compared. After controlling for patients’ characteristics, relevant variation between local health units was detected. The MOR at LHU-level was the highest in both regions and the only statistically significant feature in the Tuscany region, at 1.26 (*p* = 0.020). In Lazio, the same measure was equal to 1.23 (*p* = 0.035). However, the results of the cross-classified model presented in Table 4 show that, after including the hospital of discharge-level, the “weights” related to community-based providers decreased and lost their significance. In Lazio and Tuscany regions, the MOR associated with the LHU-level decreased to 1.09 (*p* = 0.154) and 1.11 (*p* = 0.181), respectively. Similar reductions are seen for LHDs and GPs. In summary, variation between community-based providers is no longer significant under the hypothesis that all patients were discharged from the same hospital; rather, the variability in patients’ adherence attributable to the hospital of discharge was substantially higher (MOR = 1.30 and 1.31 in both Lazio and Tuscany regions, respectively) and statistically significant (*p* = 0.001 for both regions).

**Table 3:** Multilevel results for community-based healthcare providers

Levels of healthcare system	Lazio			Tuscany		
	MOR	*p* value	95% CI	MOR	*p* value	95% CI
Local health unit	1.23	0.035	1.13–1.42	1.26	0.020	1.16–1.46
Local health district	1.12	0.018	1.08–1.20	1.12	0.089	1.06–1.26
General practitioner	1.17	0.144	1.07–1.49	1.09	0.349	1.01–3.03

MOR, median odds ratio; CI, confidence interval

**Table 4:** Cross-classified multilevel results: the impact of hospitals of discharge

Levels of healthcare system	Lazio			Tuscany		
	MOR	*p* value	95% CI	MOR	*p* value	95% CI
Hospital of discharge	1.30	0.001	1.21–1.44	1.31	0.001	1.22–1.44
Local health unit	1.09	0.154	1.03–1.26	1.11	0.181	1.04–1.35
Local health district	1.08	0.111	1.04–1.20	≈ 1.00	–	–
General practitioner	1.15	0.175	1.07–1.49	≈ 1.00	–	–

MOR, median odds ratio; CI, confidence interval

## Discussion

This study provides new insights into the drivers of adherence to best practice treatment in patients following admission to hospital with acute MI, using data from over 32,000 individuals in two Italian regions. In particular, the cross-classified model enables quantification of the impacts of different elements of health system related factors on patient adherence up to two years post-discharge from hospital. This analysis helps understand—and potentially address—the drivers of an important issue in many health systems affecting patient outcomes and cost.

### Epidemiological findings

Our findings provide evidence of the scale of non-adherence to best practice treatments following acute MI in Lazio and Tuscany regions; in Lazio only 63% patients are adherent to best practice, and in Tuscany only 59%. Patients affected by STEMI in the index admission were more adherent to medication than patients with NSTEMI. It is likely that patients with less severe symptoms perceive the benefits of medication adherence to a lesser extent, and are therefore less inclined to follow a correct therapeutic regimen. Additionally, population analysis shows that adherence is not equally distributed, with the highest levels of non-adherence among women [21], and the most elderly. Of particular note is the steep drop-off in adherence to chronic polytherapy between the second oldest and oldest age groups, with drops in adherence of at least 17 percentage points observed between these groups across both regions and sexes. This drop-off forms one end of the U-shaped curve of age against adherence, in line with the trend identified in previous studies on adherence to statins [22]. These findings indicate the presence of health inequalities within patient populations in adherence to best practice following discharge. It also indicates that models of discharge and post-discharge support are not equally efficacious at ensuring adherence to best practice across all patient groups. These results can be considered alongside previous evidence of the steep drop-off in adherence to polytherapy as a function of time following discharge [23]. Taken together, this suggests that the current models of care and support in Lazio and Tuscany do not sufficiently serve those patients with the most health and social needs, at least with respect to ensuring adherence to best practice treatment.

### Provider impact analysis

Our analysis indicates inequalities in adherence based on treatment received during hospital stays. The impact of the type of discharge ward was notable: patients discharged from cardiology wards were much more likely to be adherent to evidence-based medications. Moreover, relevant variability in adherence to guidelines was observed between healthcare providers, in both Lazio and Tuscany regions. This kind of geographic heterogeneity raises equity concerns in access to optimal care, and highlights the lack of regional shared guidelines. A strength of the cross-classified model is that it enables quantification of the impact of different providers of the healthcare system, accounting for patient characteristics. Most notably, our model demonstrates that when the hospital of discharge is included, all other community-based providers have a reduced impact on adherence to pharmacological clinical guidelines. In the cross-classified model, most of the variability in patient adherence observed between community settings is therefore explained by the hospital of discharge, up to two years after the acute event.

### Interpreting the data

Evidence on the factors affecting medication adherence among older people primarily relates to patient characteristics, rather than system factors [24], and overall, this evidence is uncertain about the factors affecting adherence to oral therapies among older people with chronic conditions (as in the patient population investigated in this paper) [25]. There is, however, evidence that follow up appointments and continuity of care can increase adherence to statins [26], and that the timing of the first follow up appointment after discharge following acute MI is associated with rates of adherence to best practice treatment [27]. In Lazio and Tuscany regions, different hospitals have different follow-up protocols addressing duration of the follow-up period and frequency of evaluations. There are also relevant differences in practice between specialist and non-specialist wards.

Based on these features and the results of our analysis, the effectiveness of the discharge process and the appropriateness of specialist follow up appointments provides the most probable explanation for the differing rates of adherence between specialist and non-specialist wards, and for the variation between hospitals. Where discharge processes are poor and follow up appointments are not properly scheduled, this may be compounded by inadequate handover to community-based health professionals, such as through discharge letters lacking sufficient information. Adherence to good practice in hospital discharge processes can therefore be seen as a major determinant of medication adherence for at least two years after discharge. Admission and bed management processes will also be relevant where this leads to patients being inappropriately discharged from non-cardiac wards following MI. There may be substantial population health gains available through a greater focus on the role of hospitals in chronic care after acute events, in particular through reducing the variation in discharge practices.

### Policy implications and actions

Our results emphasise that the hospital setting is an important driver of patient behaviours and outcomes related to chronic conditions. The role of hospitals may therefore be insufficiently represented in prevailing policy efforts to improve care for chronic conditions, the broad focus of which is typically on the role of primary and community services in supporting people, and which implicitly or explicitly seek to avoid the use of hospital-based care as far as possible. Initiatives specifically aiming to increase therapy adherence are typically of a similar focus [14]. These models are predicated on the idea that individuals with ongoing needs can be most effectively supported in the community, providing greater convenience for patients and lower cost for health systems. This may not always hold true, especially for patients discharged following a severe and acute event. For this group, there may be an important ongoing role for the hospital based medical teams in encouraging adherence to best practice treatment.

However, while challenging the current degree of focus on out-of-hospital settings for populations with ongoing care needs, our findings do not imply that community-based services should have a diminished role in the care of such patients. It is unclear how far the impact of community-based settings in supporting adherence might be increased through different ways of working e.g. a transfer of responsibilities from hospital-based specialists to community-based professionals. One reasonable response to these findings would be to redouble efforts to increase the role of community-based providers in the management of chronic patients. A balanced policy approach might combine a specific aim to increase adherence to good practice in discharge processes along with support for practical action at operational levels in health systems, including in community settings. This could both improve patient outcomes and enable a more effective use of resources through avoiding downstream negative outcomes and potential readmissions [8].

There are several levers available to support such improvements. The current DRG-based tariff for acute MI in Lazio, for example, includes the first outpatient appointment in the bundled payment, which is supported by regional guidance for hospital teams which requests first follow-up appointment scheduling within 30 days of discharge. These policy interventions are intended to bridge the acute and community-based care periods for such patients. These features are not present in Tuscany, where the DRG does not include the first outpatient appointment, thereby leaving patients liable for charges (specialist outpatient appointments are subject to co-payments in Italy; this does not apply to inpatient or primary care). However, while we do not have data comparing levels of first cardiology outpatient appointments between Lazio and Tuscany, the policy interventions in Lazio appear ineffective at increasing adherence to therapy following discharge since there are no relevant differences in adherence between the two areas. Such models can be effective with a clearer link between payment and real performance; the tariff and guidance could be adapted to better improve adherence (use of a best practice tariff was previously effective at changing hospital practices in Italy [28]). There is also evidence from Italy that performance data can be benchmarked and more effectively shared with providers at both acute and community levels, to collectively agree and deliver effective improvement actions [29]. Targeted information sharing with physicians, such as in ‘audit and feedback’ approaches, can be effective at changing physician behaviors when appropriately designed and delivered [30].

### Study strengths and limitations

Primary strengths of the study derive from the use of robust methodological procedures applied to large population-based datasets in two areas in Italy. The cross-classified model enables quantification of the impact of different elements within the healthcare system, and therefore helps enable diagnosis of challenges and supports improvement actions at policy and practice levels.

A consideration in using drugs claims databases in analysis is that, while they provide a strong source for information about prescription collection among populations in real-world settings, it is not possible to interrogate actual consumption of the collected prescriptions. Additionally, the database does not include data about prescribed daily doses, so adherence is calculated based on DDDs. However, this is a recognized method which helps enable comparisons even if some drug-use misclassification may have taken place.

## Conclusions

Adherence to best practice treatments after MI is not consistent with clinical guidelines, and varies between patient groups as well as within and between providers and sub-regions. The variation attributable to providers is almost fully accounted for by the hospital of discharge, when this is included in the cross-classified model. It is feasible that much of this variation is attributable to discharge processes, in particular through how far they support effective transitions in and continuity of care. A range of policy tools could be appropriate to reduce this variation which could lead to substantial population health gains and more appropriate resource use.

## Supplementary Information

**Additional file 1.** Appendix.

## Data Availability

The datasets used and analyzed during the current study are available from the corresponding author on reasonable request.

## Abbreviations

MI: Myocardial infarction
STEMI: ST-elevation MI
NSTEMI: Non-ST-elevation MI
NHS: National Health System
LHU: Local health unit
LHD: Local health district
GP: General practitioner
EB: Evidence based
ATC: Anatomical therapeutic chemical
MPR: Medication possession ratio
ICD-9-CM: International classification of diseases-ninth revision-clinical modification
PCI: Percutaneous coronary intervention
CABG: ‘Bypass’
MOR: Median odds ratio
OR: Odds ratio
CI: Confidence interval
