# Supplementary material for: Chronic polytherapy after myocardial infarction: the trade-off between hospital and community-based providers in determining adherence to medication
Source: BMC Cardiovasc Disord. 2021 Apr 14;21:180. doi: 10.1186/s12872-021-01969-9 (PMC8048349; doi:10.1186/s12872-021-01969-9)
Supplement: Supplementary file 1 — Additional file 1. Appendix. [file 12872_2021_1969_MOESM1_ESM.doc]

**APPENDIX**

***Data sources***

*Hospital Information System (HIS)*

The HIS includes the patients’ characteristics (the patients’ identifier, gender, date and place of birth, and place of residence); admission and discharge dates; discharge diagnoses (up to 6); procedure codes (up to 6) according to the International Classification of Disease, Ninth Revision, Clinical Modification (ICD-9-CM); hospital ward(s); date(s) of in-hospital transfer; and a regional code that corresponds to the admitting facility.

*Mortality Information System (MIS)*

The MIS includes the patients’ demographic characteristics (the patient’s identifier, age, gender, place and date of birth, residence, marital status, and occupation), as well as the date, place, and cause of death (codified by ICD-9 codes).

*Drug claims registry (Pharm)*

Pharm registry comprises individual records for each medical prescription dispensed in public and private pharmacies within the territory of the local health authorities for the resident population. The registry is limited to those drugs prescribed for outpatient use that are reimbursed by the health care system. All the drugs in the study are included in the Pharm registry. In this registry, the drugs are identified by the national drug registry code, which refers to the international ATC classification and allows for the exact quantification of the dispensed drug. Individual patient data (the patient’s identifier) and the date the drug is dispensed are reported for every prescription.

Prescriptions of antiplatelets (ATC codes: B01AC04, B01AC05, B01AC06), β-blockers (ATC code: C07), ACEI/ARBs (ATC code: C09) and statins (ATC code: C10AA) were collected.

**Algorithm for selection of the cohort**

ICD-9-CM codes for the identification of MI cases.

An MI was defined either as a primary diagnosis of an acute myocardial infarction (ICD-9-CM 410.xx) or as a secondary diagnosis of a MI associated with one of the following conditions as the primary diagnosis.

**ICD-9-CM code Condition**

411 Other acute and subacute forms of ischemic heart disease

413 Angina pectoris

414 Other forms of chronic ischemic heart disease

423.0 Haemopericardium

426 Conduction disorders

427 Cardiac dysrhythmias, excluding 427.5 Cardiac arrest

428 Heart failure

429.5 Rupture of chordae tendineae

429.6 Rupture of papillary muscle

429.71 Acquired cardiac septal defect

429.79 Certain sequelae of myocardial infarction not elsewhere classified

429.81 Other disorders of papillary muscle

518.4 Acute edema of lung, unspecified

518.81 Acute respiratory failure

780.01 Coma

780.2 Syncope and collapse

785.51 Cardiogenic shock

799.1 Respiratory arrest

997.02 Iatrogenic cerebrovascular infarction or haemorrhage

998.2 Accidental puncture or laceration during a procedure

**Selection of comorbidities from hospital discharge re**cords

| **Condition** | **ICD-9-CM diagnosis codes** | |
| --- | --- | --- |
|  | **Index admission** | **Admissions during the 9 years prior to index admission** |
| Malignant neoplasms | 140.0–208.9 | 140.0–208.9, V10 |
| Diabetes | 250.0-250.9 | 250.0-250.9 |
| Disorders of lipoid metabolism | 272 | 272 |
| Obesity | 278.0 | 278.0 |
| Anemias | 280-284, 285 (excl. 285.1) | 280-284, 285 (excl. 285.1) |
| Coagulation defects | 286 | 286 |
| Other hematologic diseases | 287-289 | 287-289 |
| Hypertension | 401-405 | 401-405 |
| Heart failure | 428 | 428 |
| Ill-defined description and complications of heart disease | 429 | 429 |
| Chronic rheumatic heart disease | 393-398 | 391, 393-398 |
| Cardiomyopathy | 425 | 425 |
| Acute endocarditis and myocarditis | 421, 422 | 421, 422 |
| Other cardiac conditions | 745, V15.1, V42.2, V43.2, V43.3, V45.0 | 745, V15.1, V42.2, V43.2, V43.3, V45.0 |
| Conduction disorders and cardiac dysrhythmias | 426, 427 | 426, 427 |
| Cerebrovascular disease | 433, 437, 438 | 430-434, 436-438 |
| Diseases of arteries, arterioles, and capillaries | 440-448 (excl 441.1, 441.3, 441.5, 441.6, 444), 557.1 | 440-448, 557 |
| Chronic obstructive pulmonary disease (COPD) | 491-492, 494, 496 | 491-492, 494, 496 |
| Chronic nephropathies | 582-583, 585-588 | 582-583, 585-588 |
| Chronic liver, pancreas, and digestive diseases | 571,572, 577.1-577.9, 555, 556 | 571-572, 577.1-577.9, 555, 556 |
| Gastro-oesophageal haemorrhage | 530.2 (2009: 530.21), 530.82, 531.0, 531.2, 531.4, 531.6, 532.0, 532.2, 533.4, 533.6, 534.0, 534.2, 534.4, 534.6, 535.01, 535.11, 535.21, 535.31, 535.41, 535.51, 578.9 | 530.2 (2009:530.21), 530.82, 531.0, 531.2, 531.4, 531.6, 532.0, 532.2, 533.4, 533.6, 534.0, 534.2, 534.4, 534.6, 535.01, 535.11, 535.21, 535.31, 535.41, 535.51, 578.9 |

| **Condition** | **ICD-9-CM diagnosis or procedures codes** |
| --- | --- |
| Other forms of ischemic heart diseases | 411, 413, 414 |
| Percutaneous coronary intervention (PCI) | 00.66, 36.01, 36.02, 36.05-36.07 |
| Coronary artery bypass graft surgery (CABG) | 36.10-36.19 |
| Other operations on heart | 35, 37.0, 37.1, 37.3-37.6, 37.9 |
| Other operation on vessels | 38-39.5 (excl. 38.01, 38.02, 38.5, 38.11, 38.12, 38.31, 38.32) |

**Selection of comorbidities and surgical procedures looked for in the index admission**
